# Introducing altmetrics to the Journal of the Medical Library Association

**DOI:** 10.5195/jmla.2017.250

**Published:** 2017-07-01

**Authors:** Katherine G. Akers

Most readers of the *Journal of the Medical Library Association (JMLA)* are well aware of the inappropriateness of evaluating individual journal articles by their journals’ impact factors. This is because, among other reasons, a journal’s citations are not evenly distributed across its articles. Rather, a small proportion (20%) of articles often accounts for most (80%) of a journal’s citations [1]. Therefore, individual journal articles deserve to be judged on their own merits.

The traditional article-level measure of impact is the number of times that an article is cited by other articles. However, article citations are slow to accrue and reflect only one dimension of the impact of one’s work: how often it is discussed in the scholarly literature. By contrast, altmetrics (“alternative metrics”) [2] provide more immediate information about reader interest as well as a broader picture of article impact. Because articles published in the *JMLA* are often more practically oriented than theoretically oriented, their impact may be better judged by the extent to which they change the practice of health sciences librarianship than by the frequency with which they are discussed in academic circles. While it may be impossible to measure the true impact of individual journal articles on a profession, altmetrics can provide insight into the influence of articles in the *JMLA* on the field of health sciences librarianship and information science by showing how frequently they are read and discussed online (Figure 1).

**Figure 1:**
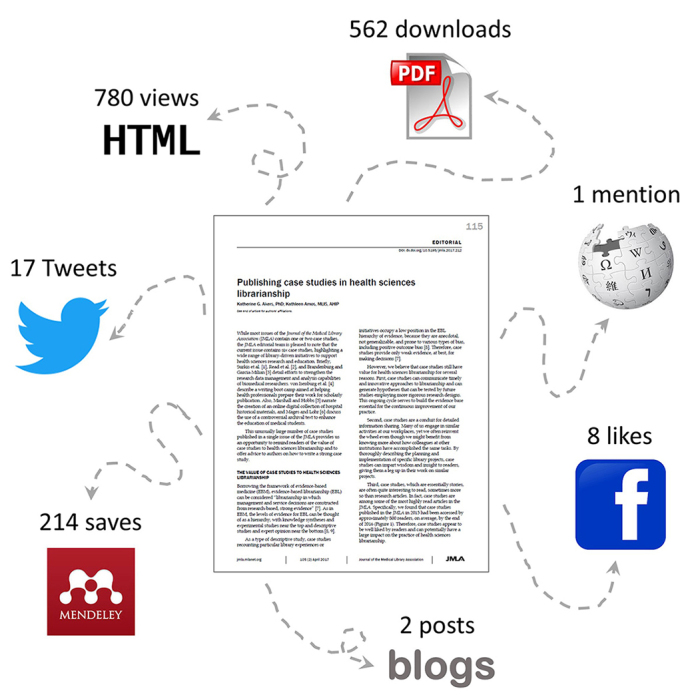
Depiction of altmetrics for a journal article

## ALTMETRICS IN THE *JOURNAL OF THE MEDICAL LIBRARY ASSOCIATION*

The *JMLA*’s new online platform makes use of PlumX, powered by Plum Analytics, to display a range of article-level metrics for each published article. PlumX divides these metrics into five categories:

- usage (e.g., abstract views, HTML views, full-text views)
- captures (e.g., Mendeley readers)
- social media (e.g., tweets; Facebook shares, likes, and comments)
- mentions (e.g., blog mentions, Reddit comments, Wikipedia links)
- citations (e.g., Scopus, CrossRef)

A look at these metrics for articles published in the most recent year of the *JMLA* (July 2016 to April 2017) reveals that many articles have been used hundreds to thousands of times and frequently mentioned in social media. The articles receiving the highest amount of social media attention to date include:

- “How Do Early Career Health Sciences Information Professionals Gain Competencies?” by Bethany A. Myers and Bredny Rodriguez [3] (106 Facebook likes, shares, and comments; 9 tweets)
- “Impact of Librarians on Reporting of the Literature Searching Component of Pediatric Systematic Reviews” by Deborah Meert, Nazi Torabi, and John Costella [4] (53 tweets)
- “Scoping Reviews: Establishing the Role of the Librarian” by Martin Morris, Jill T. Boruff, and Genevieve C. Gore [5] (39 tweets)
- “De-Duplication of Database Search Results for Systematic Reviews in EndNote” by Wichor M. Bramer and colleagues [6] (29 tweets)

## ARTICLE IMPACT ON PROFESSIONAL PRACTICE

In the biomedical sciences, basic research is cited much more frequently than clinical research [7, 8], perhaps because basic research tends to prompt further basic research and/or clinical trials that are later published, whereas clinical research tends to change clinical practice. A similar divergence might occur in the field of library and information science, with articles published in more theoretically oriented information science journals receiving more citations than articles published in more practically oriented library journals like the *JMLA.* Thus, citations may not be the best measure of impact for articles that receive attention from readers who might not frequently contribute to the scholarly literature, such as practicing librarians and library students [9, 10]. Rather, by reflecting at least part of the online conversation about particular articles, altmetrics can provide a more encompassing view of the influence of articles on society, including their professional and educational impact.

## DYNAMICS AND PREDICTIVE VALUE OF ALTMETRICS

Many studies aiming to understand the dynamics and predictive value of altmetrics have examined their temporal distribution and correlation with citations. For instance, one study reports that most tweets about an article occur in the first two days of its publication, with a plateau after five to seven days, demonstrating how quickly altmetrics reflect interest in an article [11]. Furthermore, the number of tweets is significantly predictive of the number of citations that an article will later receive [11], suggesting that mentions of an article in social media are a reasonably valid measure of its impact. However, there are notable differences in the magnitude of correlations between citations and different altmetric indicators, supporting the idea of different “flavors” of impact [12]. In general, traditional citations appear to be more strongly correlated with measures of article usage (i.e., views, downloads) and saves in social reference managers (e.g., Mendeley readers) and less strongly correlated with mentions in social media (e.g., Facebook, Twitter) or blogs [13–15].

## SELF-PROMOTION OF YOUR WORK

If you author an article in the *JMLA,* the day of its publication is undoubtedly a moment for celebration. However, we hope that your work will continue to resonate with readers long after it is published. A necessary first step, however, is to get your work into the hands (or eyes) of readers. Our team at the *JMLA* and the Medical Library Association (MLA) actively promote the contents of the *JMLA* through multiple avenues, including Twitter (@JrnlMedLibAssn), Facebook, and email announcements to MLA members and readers who are registered with the journal website. However, promoting your own work can go a long way toward drawing further attention to your article and thus expanding its audience and impact. To increase your article’s altmetrics, try the following:

- Announce your article through Twitter, Facebook, and other social media platforms.
- Post about your article on a personal and/or institutional blog.
- Deposit a copy of your article into your institutional repository.
- Add article details to your ORCID, LinkedIn, Google Scholar, or other professional profile.
- Email copies of your article to colleagues and other authors who have influenced your work.
- Talk about your article at conferences.

Finally, we encourage you to include your article’s altmetrics on your CV or professional dossier [12] to provide evidence of the impact of your work on the thinking and practice of health sciences librarians and information specialists.
